# NSCLC Digital PCR Panel Returns Low-Input Sample Results Where Sequencing Fails

**DOI:** 10.3390/diagnostics14030243

**Published:** 2024-01-24

**Authors:** Leah Rowland Herdt, Paige Berroteran, Malini Rajagopalan, Bradley A. Brown, Jerrod J. Schwartz

**Affiliations:** ChromaCode Inc., 2330 Faraday Ave, Carlsbad, CA 92008, USA

**Keywords:** NSCLC, dPCR, NGS, QNS

## Abstract

Molecular diagnostics has drastically improved the survival rate of patients diagnosed with non-small cell lung cancer (NSCLC) over the last 10 years. Despite advancements in molecular testing, targeted therapies, and national guideline recommendations, more than half of NSCLC patients in the United States either never receive testing or patient care is not informed via molecular testing. Here, we sought to explore the relationship between DNA/RNA input, the molecular testing method, and test success rates. On a shared set of low-input reference test materials (*n* = 3), we ran both a hybrid capture-based, next-generation sequencing (NGS) assay and a multiplexed digital PCR (dPCR) panel. The dPCR panel was highly sensitive and specific for low-input samples in dilution studies ranging from 40 to 1 ng DNA and from 20 to 2.5 ng RNA, while NGS had up to an 86% loss in sensitivity as contrived sample inputs were serially diluted. The dPCR panel also demonstrated a high PPA (>95%) at diluted inputs as low as 15/7.5 ng DNA/RNA on 23 banked clinical samples with the same NGS hybrid capture assay at a high input. These data suggest that digital PCR is an accurate and effective way of identifying clinically relevant NSCLC mutations at low nucleotide input and quality.

## 1. Introduction

NSCLC is the most common type of lung cancer, accounting for 85% of all lung cancer diagnoses [1]. Over the last decade, precision medicine, and its focus on treatments based on the pathomechanisms of disease in the individual patient, has ushered in the use of targeted therapies and has significantly improved patient survival rates for NSCLC [2]. The National Cancer Comprehensive Network (NCCN) guidelines currently recommend molecular testing, as part of a broad assessment and gene coverage, for identification of targeted therapies or clinical trials [3]. While precision medicine has demonstrated improved outcomes in NSCLC and other tumors, uptake in molecular testing and subsequent use of targeted therapies has lagged in implementation, particularly in the community setting, with as many as 50% of patients not obtaining testing and of those that did obtain testing, 30% may not be obtaining the most appropriate therapy [4,5,6]. The causes for this are multifactorial and not fully explored, but the concept of the lack of deployment of best-available therapeutic interventions as a result of a failure of the process of appropriate testing, communication of data, and utilization of the results is best summarized as “leakage” [5,6,7,8,9]. The clinical laboratory is not exempt from contributing to this process. Critical issues faced by the lab include the timeliness of obtaining results to ordering clinicians, the cost of validating and performing complex genomic tests, ambiguity over reimbursement, and in particular for NSCLC, often working with limited samples that have insufficient DNA or RNA for processing [5,6,7,8,9]. As a result, many labs forgo this testing and leave it to centralized reference labs, often in other states, who may also have issues with turnaround times and client services [5]. Of particular note, labs have reported sample failure rates of approximately 20.2%, likely as a result of working with small samples such as fine needle aspirate or core needle biopsy material, and performing high-input requirement procedures like next-generation sequencing (NGS) testing and, particularly, comprehensive genomic profiling (CGP) [10,11]. Approximately 20% of patients with NSCLC would benefit from targeted therapies, but because of a lack of material, failed NGS testing, or delayed results, <10% of patients receive targeted therapy [12,13].

To address these issues, we have developed a low-cost, low-complexity, rapid turnaround time test: the ChromaCode HDPCR™ NSCLC Panel, a High Definition digital PCR-based assay intended to provide NCCN guideline-directed, clinically relevant genomic information. The test is designed to work with minimal amounts of DNA and RNA, which can potentially rescue failed assays utilizing NGS methodology. A dPCR-based approach may also be utilized using local molecular testing facilities based on its low cost and greatly decrease turnaround times based on processing times. An initial trial use and content of the assay has been previously published [14].

In this study, we demonstrate the performance of the HDPCR™ NSCLC Panel in both reference and biological samples, and compare the results to a current best-in-class assay, the Illumina Trusight Oncology 500 (TSO500), clinically validated and operated in a CAP and CLIA lab, in limited DNA and RNA quantity conditions that are common to NSCLC samples. We illustrate the performance of the TSO500 and HDPCR™ at inputs below validated thresholds and its impact to variant detection. We also demonstrate the reliability of the dPCR assay in low-input conditions compared to TSO500 in validated conditions and, for the first time, provide evidence that meaningful and consistent results can be obtained for most clinically relevant biomarkers within a few hours with low-quality samples and with as little as 10 ng of DNA and 5 ng of RNA with the HDPCR™ NSCLC Panel.

## 2. Materials and Methods

### 2.1. Materials

Twenty-four anonymized, remnant human biological FFPE samples were obtained from Precision for Medicine (Frederick, MD, USA), BioChain (Newark, CA, USA), CHTN (Durham, NC, USA), or Lykos Labs (Conroe, TX, USA) and six FFPE reference standards (50% variant allele frequency) were obtained from Horizon Discovery (Cambridge, UK) (Supplemental Table S1). Lykos Lab performed the Illumina TSO500 testing on Illumina NovaSeq 6000 (San Diego, CA, USA) at 100 ng DNA and RNA input to establish the sample truth for purposes of test performance in collaboration with their partner laboratories.

### 2.2. HDPCR NSCLC Panel

The HDPCR NSCLC Panel (ChromaCode, Carlsbad, CA, USA) utilizes dPCR, where endpoint fluorescent intensities are modulated such that each unique target produces a unique endpoint intensity. The assay consists of three wells: two wells detect DNA targets, and one well detects RNA fusions. All runs were performed on the QIAcuity (Qiagen, Germany) using the QIAcuity Nanoplate 26K 24-well plate. The master mix for DNA wells was formulated by combining 10.5 µL of QIAcuity Probe Master Mix, 8.4 µL of HDPCR Mix, and varying µL of molecular-grade water per reaction. The master mix for each RNA well was formulated by combining 10.5 µL of QIAcuity OneStep Advance Probe Master Mix (Qiagen, Germany), 0.45 µL of OneStep RT Mix (Qiagen, Germany), 8.4 µL of HDPCR Mix [14], and varying µL of molecular-grade water per reaction. After preparation of the master mix, varying µL of the 1 ng/µL sample was added to the appropriate master mix and mixed thoroughly. From this mixture, 39 µL was added to a well on the QIAcuity Nanoplate. The plate then underwent thermocycling on the QIAcuity according to the instructions for use. Analysis was carried out using the ChromaCode Cloud, which reports detected targets and estimated MAF (mutant allele fraction). The estimated MAF is calculated as (target counts/internal control counts) × 100.

### 2.3. Illumina TSO500 and HDPCR NSCLC Panel Performance with Limited DNA and RNA Input

Three unique FFPE reference standards were enrolled in this study. DNA and RNA were extracted from three 10 µm curls using the Maxwell HT FFPE DNA Isolation System (Promega, Madison, WI, USA) on the KingFisher™ Flex instrument (Thermofisher, Carlsbad, CA, USA). Following extraction, eluates were quantified using the Qubit dsDNA BR Assay Kit (Invitrogen, Waltham, MA, USA) or the Qubit RNA BR Assay Kit (Invitrogen, Waltham, MA, USA). Samples were evaluated with the HDPCR NSCLC Panel using the abovementioned methods at 40, 20, 15, 10, 5, 2, 1, and 0.5 ng DNA and 20, 10, 7.5, 5, 2.5, 1, 0.5, and 0.25 ng RNA. The same extracted material was evaluated with the Illumina TSO500 NGS Assay according to the clinically validated test specifications of Lykos Labs at 100, 40, 20, 15, and 10 ng DNA and 100, 20, 10, 7.5, and 5 ng RNA, with the <100 ng inputs being below validated or recommended input quantities to simulate operations with limited samples.

### 2.4. HDPCR NSCLC Panel Low Input Concordance with TSO500

Twenty-seven unique FFPE blocks were enrolled in this study: twenty-four were biobanked anonymized tissue samples and three were FFPE reference standards. DNA and RNA were extracted from three 10 µm curls using the Maxwell HT FFPE DNA Isolation System (Promega, Madison, WI, USA) on the KingFisher™ Flex instrument (Thermofisher, Carlsbad, CA, USA). Following extraction, eluates were quantified using the Qubit dsDNA BR Assay Kit (Invitrogen, Waltham, MA, USA) or the Qubit RNA BR Assay Kit (Invitrogen, Waltham, MA, USA). Samples were evaluated with the HDPCR NSCLC Panel using the abovementioned methods at 40, 20, 15, and 10 ng DNA and 20, 10, 7.5, and 5 ng RNA. The same extracted material was evaluated with the TSO500 NGS Assay at 100 ng according to the clinically validated procedures of Lykos Labs.

## 3. Results

### 3.1. General Sample Performance and Quality Metrics

All 30 enrolled samples across both studies were processed on both the Illumina TSO500 assay and the HDPCR NSCLC Panel at recommended inputs. All samples were sequenced on the TSO500 at 100 ng input to identify expected clinical variants for orthogonal comparison (Table 1). DIN (DNA Integrity Number) and RIN (RNA Integrity Number) provide information regarding the quality of gDNA and RNA, respectively. As DNA and RNA degrade, DIN and RIN decrease; therefore, it is recommended that DIN > 7 and RIN > 8 [15]. Several of the samples used in this study had suboptimal quality as measured using DIN or RIN (Table 1) but still yielded acceptable results. However, one DNA sample (CC00-02) did not meet quality requirements, was sequenced, and failed to generate data. This sample was removed from subsequent analysis. One poor-quality RNA sample (CC00-08) had no results generated from sequencing and this sample was removed from the rest of this study.

### 3.2. Illumina TSO500 Performance with Limited DNA and RNA Input Compared to the HDPCR NSCLC Panel

Illumina has a preanalytical QC criteria input requirement of 40 ng RNA and DNA. According to the manufacturer’s instructions for the TSO500 assay, DNA samples are recommended to have a coverage of a minimum of 81.3% of exonic bases with greater than or equal to 250× unique read coverage; it is likely that using less than the 40 ng minimum input precludes meeting these criteria. The clinical lab performing the validated NGS assay has an established cutoff of 50 variant supporting reads for reporting DNA variants and a sample input of 80 ng, because the internal lab validation demonstrated that there was a decrease in assay performance from 80 ng to 40 ng.

To test the effects on clinical variant detection and reporting with nucleotide inputs below this threshold, we performed the assay with contrived reference samples with known, clinically relevant mutations (at 50% variant allelic frequency) with DNA and RNA inputs at 40, 20, 15, and 10 ng of DNA and 20, 10, 7.5, and 5 ng of RNA.

The TSO500 assay, as expected, demonstrates poor performance as the input is decreased from the initial 100 ng DNA and RNA inputs. General sample metrics significantly degrade as input nucleotide quantities are decreased (Table 2). The positive percent agreement (PPA) for DNA variants of the TSO500 panel at lower inputs to the standard input of 100 ng was 75% at 40 ng, 50% at 10 and 20 ng, and 25% at 10 ng DNA. The PPA of RNA variants of the TSO500 panel at lower inputs was 33% at 20 ng, 67% at 10, and 0% at 7.5 and 5 ng RNA (Table 3).

The mean variant supporting reads on the TSO500 assay for clinically relevant variants at low inputs was less than 50 reads for all samples (3/3) tested at 15 ng or less and was under 50 reads for one sample (1/3) at 20 ng input (Supplemental Figure S1), inferring that these variants would not likely be detected and reported.

The same dilutions and additional inputs of 2, 1, and 0.5 ng for DNA and 1, 0.5, and 0.25 ng for RNA were tested on the HDPCR NSCLC Panel. Post-PCR internal controls (Supplemental Table S2) were used to identify quantity insufficient (QNS) samples, and the HDPCR NSCLC Panel had a 0% panel QNS rate for all sample inputs of 40 to 2 ng DNA and 20 to 1 ng RNA (Table 4). When the serial dilutions were performed on the HDPCR NSCLC Panel down to 0.5 ng DNA and 0.25 ng RNA, PPA and accuracy was maintained for all samples above 1 ng DNA and 2.5 ng RNA (Table 5). These results suggest that the HDPCR NSCLC Panel can be used to accurately detect variants with inputs as low as 1 ng DNA and 2.5 ng RNA, even when the DNA and RNA quality (as measured using DIN and RIN) are subpar.

### 3.3. HDPCR NSCLC Performance on Reference and Biological Samples at Low Input

Twenty-four banked clinical and three reference samples (not tested above) were sequenced on the Illumina TSO500 assay under validated conditions and then run on the HDPCR NSCLC Panel at low inputs to demonstrate the test performance below accepted NGS input requirements. Reports were generated for samples that passed sample quality criteria (IC) (Table 6). This included 27 of 27 (100%) DNA samples at 40 and 20 ng input, 26 of 27 (96.2%) DNA samples at 15 ng input, and 24 of 27 (88.9%) DNA samples at 10 ng input. Reports were generated for 27 of 27 (100%) RNA samples at 20 ng input, 26 of 27 (96.2%) RNA samples at 10 ng input, 25 of 27 (92.6%) RNA samples at 7.5 ng input, and 18 of 27 (66.7%) RNA samples at 5 ng input. Internal control quality data was highly correlated to DIN and RIN scores (Supplemental Figures S2 and S3). There were no false positive calls made at any input.

In samples that passed internal control quality criteria (down to 10 ng input), the performance of the HDPCR NSCLC Panel compared with TSO500 results was excellent (Table 7). For DNA, the PPA for individual targets was 100.0%, and the positive predictive value (PPV) was 100% when the target was present. For RNA, at 20 and 7.5 ng input, the PPA was 100%, 86% at 10 ng input, and 50% at 5 ng input (Table 7). The PPA for individual targets was 50–100.0%, and the PPV was 100% when the target was present for samples above 7.5 ng. The negative predictive agreement (NPA) was 100.0%, and the negative predictive value (NPV) was >99% for samples above 7.5 ng (Supplemental Table S3).

These data demonstrate that at low inputs, using banked clinical samples, the HDPCR NSCLC Panel provides accurate results down to 10 ng DNA with only a 6% QNS rate, and the RNA is accurate with a QNS rate down to 8% at 7.5 ng. The test performed much better with contrived samples (Supplemental Table S4), which may be associated with the relatively worse DIN and RIN scores of the banked clinical samples (Supplemental Table S3).

## 4. Discussion

Despite advancement in novel therapies and diagnostic testing for NSCLC patients, key hurdles must still be overcome to best manage patients. One limitation of the current standard-of-care tests using next-generation sequencing is the requirement for large quantities of high-quality DNA and RNA, resulting in many patients receiving QNS results or requiring repeat biopsies. The results of the studies presented here illustrate a problem for NGS-based tests: a decrease in the sample quality and input severely diminishes the performance of the test and justifies the high sample quality and quantity requirements. This is largely due to inefficiencies in the sequencing library preparation, whereby molecules input into the process are lost during ligation, hybrid capture, or library dilution steps.

We also demonstrate that the HDPCR NSCLC Panel can be used to rescue findings in many samples of low quality and quantity. NSCLC-specific biomarkers had up to an 86% loss in sensitivity as inputs were serially diluted on TSO500 (43% loss in sensitivity at 40/20 and 20/10 ng DNA/RNA, a 71% loss in sensitivity at 15 ng/7.5 ng DNA/RNA, and an 86% loss in sensitivity at 10/5 ng DNA/RNA as compared to 100 ng TSO500 results using contrived samples). This loss in sensitivity could, in part, be due to the diminished quality of the samples. In contrast, the HDPCR NSCLC was 100% accurate and sensitive for the same samples at 40, 20, 15, 10, 5, 2, and 1 ng DNA and 20, 10, 7.5, 5, and 2.5 ng RNA. At all DNA inputs, there is no loss in accuracy or sensitivity, and at the lowest input of 10 ng DNA, there is only a 13% QNS rate for a complete DNA panel and 6% for a partial panel. Similar performance was seen in banked clinical samples at low inputs and low-quality scores. The HDPCR NSCLC Panel demonstrated a high PPA (>95%) with the TSO500 assay at inputs as low as 15/7.5 ng DNA/RNA. Taken together, the results demonstrate how the HDPCR NSCLC Panel can test for NCCN guideline-directed, clinically relevant variants with low nucleic acid input when NGS methods are unable to. The ability to provide accurate results for these biomarkers at low mass input is a crucial step toward rescuing samples below the limit of detection for NGS.

One of the advantages of the HDPCR NSCLC panel is that DNA targets are split across two wells; therefore, partial DNA results can still be reported if one of the two wells passes the internal control requirement. Additionally, the test benefits from minimal process handling time, and a significantly lower cost than NGS testing.

The HDPCR NSCLC Panel provides information about the most relevant clinical variants needed to make immediate decisions about patient management. Unlike TSO500 (523 genes), the HDPCR NSCLC Panel is not a comprehensive genomic profile and should not be considered a substitute for one. However, it is reasonable to foresee how such a test could improve patient outcomes when properly utilized. For example, clinicians could consider performing this simple, rapid assay locally and immediately upon diagnosis from needle core or bronchial biopsies, which are among the common sample types obtained for NSCLC diagnosis and the most frequent samples to result in a CGP test failure. The test yields NCCN-informed results for 15 NSCLC variants across nine genes, representing ~25 targeted treatment modalities, such as a *KRAS* G12C or *EGFR* L858R mutation, which could initiate adagrasib or erlotinib immediately rather than waiting for several weeks or more to find the same information from a CGP assay. If the test is negative, a CGP could be ordered because it would provide other relevant information not captured by this assay. Of note, this assay is for research use only (RUO) and requires clinical validation and testing in a clinical setting. Additional studies will determine the analytical and clinical validity of the test in such a setting, including a measurement of the improvement of failed tests in a clinical laboratory setting. We demonstrated that the HDPCR NSCLC Panel has >95% PPA with the TSO500 assay with inputs as low as 15ng DNA and 7.5 ng RNA. These data strongly support the use of the HDPCR NSCLC Panel as an accurate and effective way of identifying clinically relevant NSCLC mutations at low nucleotide input and quality.

## Figures and Tables

**Table 1:** Agilent TapeStation sample quality metrics. Results listed are only the shared variants across the TSO500 and HDPCR NSCLC Panel.

ID	TSO 500 Result (100 ng)	Source	DIN	RIN
CC00-01	ALK Fusion	Lung	1.9	2.1
CC00-02 ^2^	ALK Fusion	Lung	2	2
CC00-04	EGFR exon 19 Deletion	Lung	2	1.3
CC00-05	EGFR S768I, EGFR G719X	Lung	2.6	1
CC00-06	EGFR exon 20 Insertion	Lung	2.1	2.8
CC00-07	EGFR exon 20 Insertion	Lung	2.1	1.3
CC00-08 ^3^	KRAS G12C	Lung	1.5	2
CC00-09	EGFR S768I, EGFR L858R	Lung	2.6	1.2
CC00-10	EGFR T790M, EGFR L858R	Lung	2.2	1.7
CC00-11	EGFR L858R	Lung	2.4	1.7
CC00-14	MET exon14 skipping	Lung	3.4	1.6
CC00-15	RET Fusion	Lung	2.1	1.2
CC00-16	RET Fusion	Thyroid	2.3	1.1
CC00-17	ROS Fusion	Lung	2.9	1.5
CC00-18	ROS Fusion	Lung	2.3	2.6
CC00-19	EGFR S768I, EGFR G719X	Lung	1.9	2
CC00-20	EGFR T790M, BRAF V600E	Horizon Discovery Reference	7.2	1
CC00-21 ^1^	EGFR L858R, BRAF V600E	Horizon Discovery Reference	6.9	1.3
CC00-22 ^1^	ALK, RET, ROS Fusion	Horizon Discovery Reference	6.5	2.7
CC00-23 ^1^	EGFR G719X, KRAS G12C	Horizon Discovery Reference	6.8	2.3
CC00-24	EGFR S768I, BRAF V600E	Horizon Discovery Reference	6.8	1.5
CC00-25	EGFR exon 19 Deletion, BRAF V600E	Horizon Discovery Reference	7.3	1
CC00-N2	none	Lung	3.4	1.2
CC00-N4	none	Lung	5.6	1.1
CC00-N5	none	Lung	5	1
CC00-N6	none	Lung	6.4	1.6
CC00-N7	none	Lung	3.7	1
L-1242 ^4^	none	Tonsil	N/A	N/A
L-1243 ^4^	none	Appendix	N/A	N/A
L-1244 ^4^	none	Tonsil	N/A	N/A

^1^ Samples included in the Illumina TSO500 performance with limited DNA and RNA input compared to HDPCR. ^2^ DNA failed to generate data. ^3^ RNA failed to generate data. ^4^ Samples were QC on Bioanalyzer and met the requirements for sequencing.

**Table 2:** Quality metrics for Illumina TSO 500 at limited inputs. DNA metrics: Percent target DNA 250× (% tgt DNA 250×) is the percent target bases with greater than 250× coverage, Percent target DNA 100× (% tgt DNA 100×) is the percent target bases with greater than 100× coverage, Mean target coverage (mean tgt coverage) is the mean depth across all the unique loci defined in the manifest file, Percent exon 100× (% exon 100×) is the percentage of exonic bases in gVCF that have greater than or equal to 100× coverage, Percent exon 50× (% exon 50×) is the percentage of exonic bases in gVCF that have greater than or equal to 50× coverage, Percent aligned reads (% aligned reads) is the percentage of reads that are mapped. RNA metrics: Percent on target (% on target) is the percentage of reads that cross any part of the target region out of the total number of reads. A read that partially maps to a target region is counted as on target, Median insert size (med insert size) is the median of fragment sizes (base pairs (bp)) calculated from read alignments, recommended threshold is ≥80 bp. Scaled median gene coverage (scaled med gene cov) is the median of median gene coverage scaled by gene length. Median coverage is calculated for each gene, and then scaled by the respective gene’s length, and finally divided by the total length of all targeted genes. Total on target reads (on target reads) is the total number of reads that map to the target regions, recommended threshold is ≥9,000,000 counts.

			DNA						RNA			
Sample	DNA Input (ng)	RNA Input (ng)	% tgt DNA 250×	% tgt DNA 100×	Mean tgt Coverage (Count)	% Exon 100×	% Exon 50×	% Aligned Reads	% on Target	Med Insert Size (bp)	Scaled Med Gene Cov	On Target Reads (Count)
CC00-21	100	100	85.1	94.2	1506	96.3	99.3	99.2	92.6	199	6030.8	27,788,858
CC00-21	40	20	59.1	77.7	355	80.7	89.1	99.1	84.4	173	2862.8	12,800,331
CC00-21	20	10	0.3	27.5	48	31.8	54	97.5	85.6	178	2804.8	12,408,443
CC00-21	15	7.5	0	1.9	26	2.2	34.5	89	61.7	151	1067.6	4,787,472
CC00-21	10	5	0	0	16.1	0	3.3	64.4	9	152	401.6	1,844,136
CC00-22	100	100	93.1	98.7	723	99.2	99.5	99.2	89.4	185	5662.9	26,828,621
CC00-22	40	20	49.2	88.3	286.8	90.3	99.1	98.5	74.1	164	465.1	2,229,858
CC00-22	20	10	0.1	2.8	39.7	3.1	37.8	93.2	87.6	150	3088.4	13,997,679
CC00-22	15	7.5	0	0.2	17.4	0.1	3.4	87.2	84.6	155	2300.1	10,628,015
CC00-22	10	5	0	0.1	14.3	0.1	0.8	90.7	79	160	1079	4,989,012
CC00-23	100	100	79.8	96.3	1114	97.2	99.5	99.2	93.9	166	5551.1	28,160,486
CC00-23	40	20	58	74.9	449.2	76.8	87.9	83.3	85	145	1776.6	7,640,876
CC00-23	20	10	5.4	41.8	100.1	46.5	60.6	84.5	58	154	1243.5	5,511,670
CC00-23	15	7.5	0	22.1	53.1	25.4	48.3	25.9	32	151	395.7	1,774,675
CC00-23	10	5	0	0	17.4	0	5.7	22.2	35.3	137	213.6	1,003,487

**Table 3:** Contrived reference sample concordance data reported by target for TSO500 at low inputs vs. TSO500 Assay at 100 ng input. Samples used included CC-0021, CC-0022, and CC-0023. The HDPCR NSCLC Panel evaluates each sample for 10 clinically relevant mutations (published in Cabrera, et al., 2023 [14]) True Positive (TP), True Negative (TN), False Positive (FP), False Negative (FN) Positive Percent Agreement (PPA), Positive Predictive Value (PPV), Negative Predictive Value (NPV), and Negative Percent Agreement (NPA). The cutoffs used for TSO500 were 50 reads DNA and 10 reads RNA, as validated. Count cutoffs for the HDPCR NSCLC Panel are listed in Table S2.

Input	TP	TN	FP	FN	Accuracy	PPA	NPA	PPV	NPV
DNA									
40 ng	3	26	0	1 ^a^	0.97	0.75	1.00	1.00	0.96
20 ng	2	26	0	2 ^b^	0.93	0.50	1.00	1.00	0.93
15 ng	2	26	0	2 ^c^	0.93	0.50	1.00	1.00	0.93
10 ng	1	26	0	3 ^d^	0.90	0.25	1.00	1.00	0.90
RNA									
20 ng	1	12	0	2 ^e^	0.87	0.33	1.00	1.00	0.86
10 ng	2	12	0	1 ^f^	0.93	0.67	1.00	1.00	0.92
7.5 ng	0	12	0	3 ^g^	0.80	0.00	1.00	1.00	0.80
5 ng	0	12	0	3 ^h^	0.80	0.00	1.00	1.00	0.80

^a^ CC00-21 BRAF V600E—detected in 39 reads; would not likely have been reported ^b^ CC00-21 BRAF V600E—detected 3 reads and CC00-23 KRAS G12C—47 reads. ^c^ CC00-21 BRAF V600E—0 reads and CC00-23 KRAS G12C—17 reads. ^d^ CC00-21 BRAF V600E—0 reads, EGFR L858R—24 reads and CC00-23 KRAS G12C—3 reads. ^e^ CC00-22 ALK fusion- 0 reads, RET fusion—7 reads. ^f^ CC00-22 ALK fusion—8 reads. ^g^ CC00-22 ALK fusion—0 reads, ROS1 fusion—6 reads, RET fusion—7 reads. ^h^ CC00-22 ALK fusion—0 reads, ROS1 fusion—8 reads, RET fusion—0 reads.

**Table 4:** Contrived reference sample QNS rate by sample type (DNA or RNA) and complete panel. Samples used included CC-0021, CC-0022, and CC-0023. DNA and RNA wells passing meet the IC requirements for the HDPCR NSCLC Panel (50 IC counts Well 1/Well 3 and 100 IC counts Well 2). DNA samples are tested across two wells (Well 1 and Well 2). DNA panel passing samples passed the IC requirements for both DNA wells. DNA Panel QNS was calculated based on samples that would receive an incomplete DNA report. Complete panel passing samples passed the IC requirements for all three wells. Panel QNS was calculated based on samples that would receive an incomplete report. All samples in this study received at least a partial report at each input, with no samples failing IC requirements for all three wells.

Input (DNA/RNA)	Complete DNA Panel Passed QC	DNA Panel QNS	RNA Wells Passed QC	RNA QNS	Complete Panel Passed QC	Panel QNS
40/20 ng	3/3	0%	3/3	0%	3/3	0%
20/10 ng	3/3	0%	3/3	0%	3/3	0%
15/7.5 ng	3/3	0%	3/3	0%	3/3	0%
10/5 ng	3/3	0%	3/3	0%	3/3	0%
5/2.5 ng	3/3	0%	3/3	0%	3/3	0%
2/1 ng	3/3	0%	3/3	0%	3/3	0%
1/0.5 ng	1/3	67%	3/3	0%	1/3	67%
0.5/0.25 ng	0/3	100%	3/3	0%	0/3	100%

**Table 5:** Contrived concordance data reported by target for the HDPCR NSCLC Panel at low inputs vs. TSO500 Assay at 100 ng input. Samples used included CC-0021, CC-0022, and CC-0023. The HDPCR test evaluates each sample for 10 clinically relevant mutations (published in Cabrera, et al., 2023 [14]) True Positive (TP), True Negative (TN), False Positive (FP), False Negative (FN), Positive Percent Agreement (PPA), Positive Predictive Value (PPV), Negative Predictive Value (NPV), and Negative Percent Agreement (NPA). The cutoffs used for TSO500 were 50 reads DNA and 10 reads RNA, as validated. Count cutoffs for HDPCR are listed in Table S2.

Input	TP	TN	FP	FN	Accuracy	PPA	NPA	PPV	NPV
DNA									
40 ng	4	26	0	0	1.00	1.00	1.00	1.00	1.00
20 ng	4	26	0	0	1.00	1.00	1.00	1.00	1.00
15 ng	4	26	0	0	1.00	1.00	1.00	1.00	1.00
10 ng	4	26	0	0	1.00	1.00	1.00	1.00	1.00
5 ng	4	26	0	0	1.00	1.00	1.00	1.00	1.00
2 ng	4	26	0	0	1.00	1.00	1.00	1.00	1.00
1 ng	4	26	0	0	1.00	1.00	1.00	1.00	1.00
0.5 ng	2	26	0	2 ^a^	0.93	0.50	1.00	1.00	0.93
RNA									
20 ng	3	12	0	0	1.00	1.00	1.00	1.00	1.00
10 ng	3	12	0	0	1.00	1.00	1.00	1.00	1.00
7.5 ng	3	12	0	0	1.00	1.00	1.00	1.00	1.00
5 ng	3	12	0	0	1.00	1.00	1.00	1.00	1.00
2.5 ng	3	12	0	0	1.00	1.00	1.00	1.00	1.00
1 ng	2	12	0	1	0.93	0.67	1.00	1.00	0.92
0.5 ng	2	12	0	1	0.93	0.67	1.00	1.00	0.92
0.25 ng	1	12	0	2	0.87	0.33	1.00	1.00	0.86

^a^ CC00-21 BRAF V600E—detected but IC Fail, results excluded, CC00-23 KRAS G12C and EGFR G719—detected but IC Fail, results excluded.

**Table 6:** Reference and Biological Sample QNS Rate for the HDPCR NSCLC Panel by sample type (DNA or RNA) and complete panel. DNA and RNA wells passing meet the IC requirements (50 IC counts Well 1/Well 3 and 100 IC counts Well 2). DNA samples are tested across two wells (Well 1 and Well 2). DNA panel passing samples passed the IC requirements for both DNA wells. DNA Panel QNS was calculated based on samples that would receive an incomplete DNA report. Complete panel passing samples passed the IC requirements for all three wells. Panel QNS was calculated based on samples that would receive an incomplete report. All samples in this study received at least a partial report at each input, with no samples failing IC requirements for all three wells.

Input (DNA/RNA)	Partial DNA Panel Passed QC	Partial DNA Panel QNS	Complete DNA Panel Passed QC	DNA Panel QNS	RNA Wells Passed QC	RNA QNS	Complete Panel Passed QC	Panel QNS
Reference Standards								
40/20 ng	6/6	0%	3/3	0%	3/3	0%	3/3	0%
20/10 ng	6/6	0%	3/3	0%	3/3	0%	3/3	0%
15/7.5 ng	6/6	0%	3/3	0%	3/3	0%	3/3	0%
10/5 ng	6/6	0%	3/3	0%	2/3	33.3%	2/3	33.3%
Biological Samples								
40/20 ng	48/48	0%	24/24	0%	24/24	0%	24/24	0%
20/10 ng	48/48	0%	24/24	0%	23/24	4%	23/24	4%
15/7.5 ng	47/48	2%	23/24	4%	22/24	8%	21/24	13%
10/5 ng	45/48	6%	21/24	13%	16/24	34%	14/24	41%

**Table 7:** Biological concordance data reported for the TSO500 Assay at 100 ng input vs. the HDPCR NSCLC Panel for DNA and RNA. True Positive (TP), True Negative (TN), False Positive (FP), False Negative (FN), Positive Percent Agreement (PPA), Positive Predictive Value (PPV), Negative Predictive Value (NPV), and Negative Percent Agreement (NPA). Count cutoffs for HDPCR are listed in Table S2. Wells excluded did not meet the IC requirements for NSCLC HDPCR (50 IC counts Well 1/Well 3 and 100 IC counts Well 2). DNA samples are tested across two wells, with five targets in each well (Well 1 and Well 2). One DNA sample and one RNA sample did not receive a TSO500 result and were removed from further analysis.

**DNA**										
**Input**	**TP**	**TN**	**FP**	**FN**	**Excluded**	**Accuracy**	**PPA**	**NPA**	**PPV**	**NPV**
40 ng	13	217	0	0	0	1.00	1.00	1.00	1.00	1.00
20 ng	13	217	0	0	0	1.00	1.00	1.00	1.00	1.00
15 ng	12	213	0	0	5 (1 Sample)	1.00	1.00	1.00	1.00	1.00
10 ng	13	202	0	0	15 (3 Samples)	1.00	1.00	1.00	1.00	1.00
**RNA**										
**Input**	**TP**	**TN**	**FP**	**FN**	**Excluded**	**Accuracy**	**PPA**	**NPA**	**PPV**	**NPV**
20 ng	7	108	0	0	0	1.00	1.00	1.00	1.00	1.00
10 ng	6	103	0	1	5 (1 Sample)	0.99	0.86	1.00	1.00	0.99
7.5 ng	7	98	0	0	10 (2 Samples)	1.00	1.00	1.00	1.00	1.00
5 ng	2	71	0	2	40 (8 Samples)	0.97	0.50	1.00	1.00	0.97

## Data Availability

The raw digital PCR data that support the findings of this study are available from the corresponding author [lherdt@chromacode.com or jschwartz@chromacode.com] upon reasonable request.

## Supplementary Materials

The following supporting information can be downloaded at: https://www.mdpi.com/article/10.3390/diagnostics14030243/s1, Table S1. Sample Demographics, Table S2. Established cutoffs for each well in the HDPCR NSCLC Panel, Figure S1. Supporting Reads on NGS, Figure S2. IC counts vs. DIN at varying inputs for reference and biological samples, Figure S3. IC counts vs. RIN at varying inputs for reference and biological samples, Table S3. Reference and biological specimen concordance data reported by target for the TSO500 Assay at 100 ng input and the HDPCR NSCLC Panel for each target, Table S4. Contrived reference sample concordance data reported for the TSO500 Assay at 100 ng input vs. the HDPCR NSCLC Panel for DNA and RNA, Table S5. Institutional Review Board Statement.
