# Supplementary material for: NSCLC Digital PCR Panel Returns Low-Input Sample Results Where Sequencing Fails
Source: Diagnostics (Basel). 2024 Jan 24;14(3):243. doi: 10.3390/diagnostics14030243 (PMC10854965; doi:10.3390/diagnostics14030243)
Supplement: Supplementary file 1 [file diagnostics-14-00243-s001.zip › diagnostics-2814621-supplementary.pdf]

diagnostics

Table S1. Sample Demographics

ID	TSO 500 Result	Source	Nucleated Cells per slide	Tissue Surface Area (mm2)	Tumor percent	Age	Gender	DIN	RIN
CC00-01	ALK Fusion	Lung	> 5000	162	60	58	M	1.9	2.1
CC00-02	ALK Fusion	Lung	3500	66	65	67	F	2	2
CC00-04	EGFR exon 19 Deletion	Lung	30000	150	100	66	M	2	1.3
CC00-05	EGFR S768I, EGFR G719X	Lung	> 5000	85	85	67	F	2.6	1
CC00-06	EGFR exon 20 Insertion	Lung	Unknown	Unknown	Unknown	35	M	2.1	2.8
CC00-07	EGFR exon 20 Insertion	Lung	Unknown	Unknown	Unknown	35	M	2.1	1.3
CC00-08	KRAS G12C	Lung	3500	55	75	68	M	1.5	2
CC00-09	EGFR S768I, EGFR L858R	Lung	> 5000	220	80	41	F	2.6	1.2
CC00-10	EGFR T790M, EGFR L858R	Lung	2500	72	50	69	M	2.2	1.7
CC00-11	EGFR L858R	Lung	2500	48	50	68	F	2.4	1.7
CC00-14	MET exon14 skipping	Lung	2500	91	65	66	M	3.4	1.6
CC00-15	RET Fusion	Lung	> 5000	30	95	Unknown	M	2.1	1.2
CC00-16	RET Fusion	Thyroid	> 5000	96	70	32	F	2.3	1.1
CC00-17	ROS Fusion	Lung	> 5000	72	80	69	M	2.9	1.5
CC00-18	ROS Fusion	Lung	> 5000	48	80	54	F	2.3	2.6
CC00-19	EGFR S768I, EGFR G719X	Lung	3000	150	80	56	M	1.9	2
CC00-20	EGFR T790M, BRAF V600E	HD Reference	N/A	N/A	N/A	N/A	N/A	7.2	1
CC00-21	EGFR L858R, BRAF V600E	HD Reference	N/A	N/A	N/A	N/A	N/A	6.9	1.3
CC00-22	ALK, RET, ROS Fusion	HD Reference	N/A	N/A	N/A	N/A	N/A	6.5	2.7
CC00-23	EGFR G719X, KRAS G12C	HD Reference	N/A	N/A	N/A	N/A	N/A	6.8	3.1
CC00-24	EGFR S768I, BRAF V600E	HD Reference	N/A	N/A	N/A	N/A	N/A	6.8	1.5
CC00-25	EGFR exon 19 Deletion, BRAF V600E	HD Reference	N/A	N/A	N/A	N/A	N/A	7.3	1
CC00-N2	none	Lung	Unknown	Unknown	0	79	F	3.4	1.2
CC00-N4	none	Lung	Unknown	Unknown	0	61	F	5.6	1.1
CC00-N5	none	Lung	Unknown	Unknown	0	70	F	5	1
CC00-N6	none	Lung	Unknown	Unknown	0	72	F	6.4	1.6
CC00-N7	none	Lung	Unknown	Unknown	0	34	M	3.7	1
L-1242	none	Tonsil	Unknown	234	0	42	M	N/A	N/A
L-1243	none	Appendix	Unknown	72	0	55	M	N/A	N/A
L-1244	none	Tonsil	Unknown	150	0	36	F	N/A	N/A

Table S2. **Established cutoffs for each well in the HDPCR NSCLC Panel.**

Target	Counts
DNA Well 1	
EGFR EXON 19 (Internal Control)	50
EGFR Exon 19 Deletion (EGFR DEL)	15
EGFR S768I	20
EGFR L858R	20
BRAF V600E	10
EGFR T790M	10
DNA Well 2	
EGFR EXON 20 (Internal Control)	100
KRAS G12C	10
EGFR L861Q	10
ERBB2 Exon 20 Insertions (ERBB2)	10
GFR G719	20
EGFR Exon 20 Insertions (EGFR INS)	40
RNA Well	
HPRT1 (Internal Control)	50
ALK	15
ROS1	40
RET	15
NTRK	50
MET	40

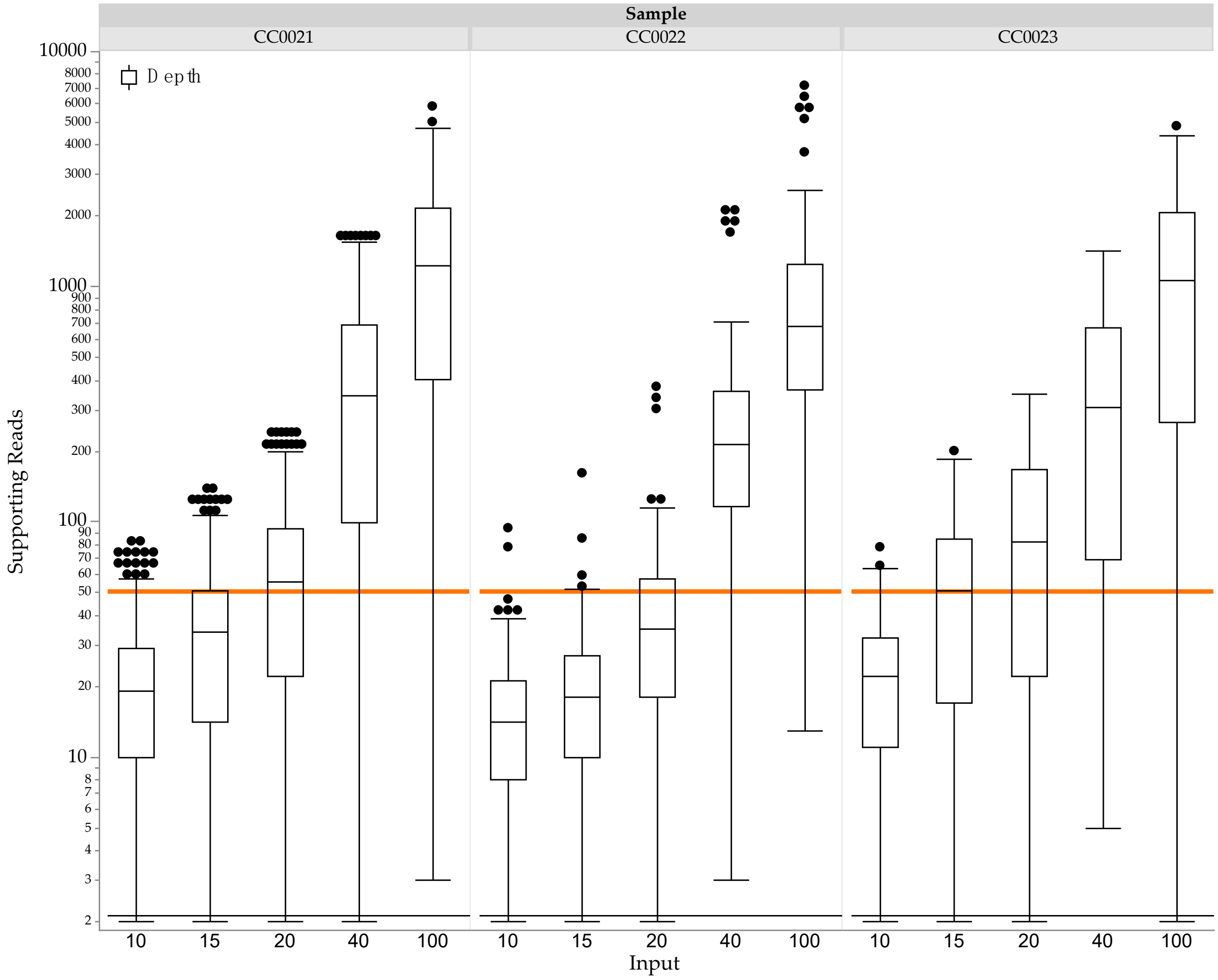

Figure S1. **Supporting Reads on NGS**. Horizon Reference Control samples (n=3). Supporting reads for the alternative allele by input (ng) across 438 genes. 50 alternative reads DNA (orange line) were the cutoffs used for TSO500.

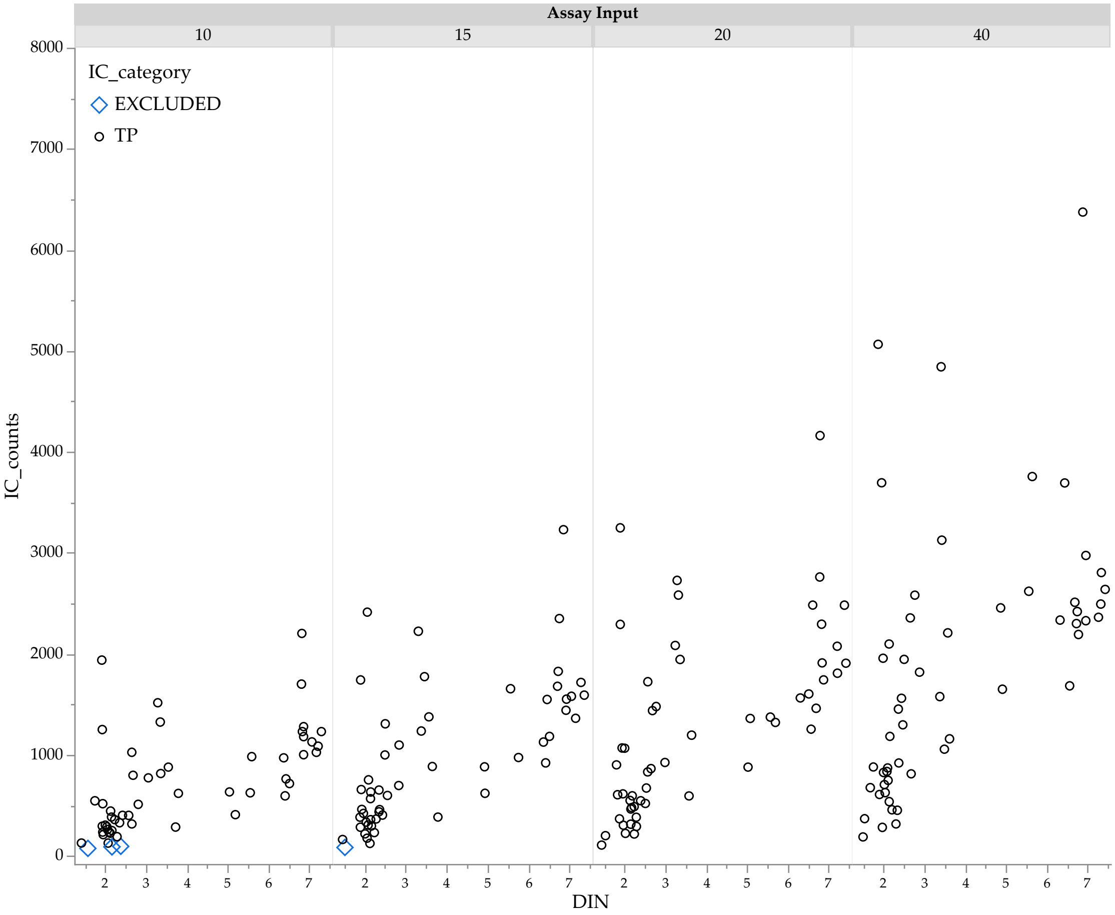

Figure S2. **IC counts vs. DIN at varying inputs for reference and biological samples**. Blue diamonds represent samples that were excluded from further analysis because they were below the IC threshold for DNA.

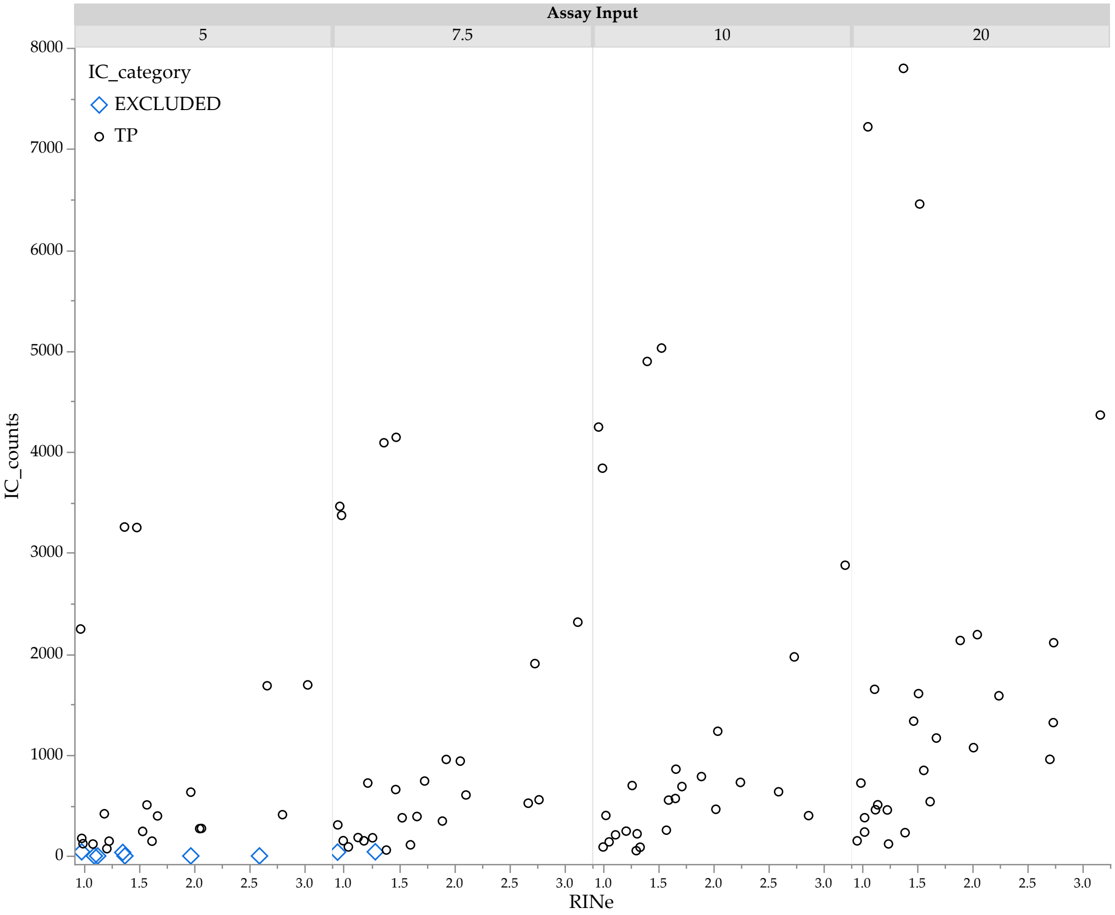

Figure S3. **IC counts vs. RIN at varying inputs for reference and biological samples**. Blue diamonds represent samples that were excluded from further analysis because they were below the IC threshold for DNA.

Table S3. **Reference and biological specimen concordance data reported by target for the TSO500 Assay at 100 ng input and the HDPCR NSCLC Panel for each target.** True Positive (TP), True Negative (TN), False Positive (FP), False Negative (FN), Positive Percent Agreement (PPA), Positive Predictive Value (PPV), Negative Predictive Value (NPV), and Negative Percent Agreement (NPA). Count cutoffs for HDPCR are listed in Table S2. Wells excluded did not meet the IC requirements for NSCLC HDPCR (50 IC counts Well 1/Well 3 and 100 IC counts Well 2). One DNA sample and one RNA sample did not receive a TSO500 result and were removed from further analysis.

	40/20 ng																			
	Reference										Biological									
call	TP	TN	FP	FN	Excluded	Accuracy	PPA	NPA	PPV	NPV	TP	TN	FP	FN	Excluded	Accuracy	PPA	NPA	PPV	NPV
ALK	1	5	0	0	0	1.00	1.00	1.00	1.00	1.00	2	21	0	0	0	1.00	1.00	1.00	1.00	1.00
BRAF V600E	4	2	0	0	0	1.00	1.00	1.00	1.00	1.00	0	23	0	0	0	1.00	-	1.00	-	1.00
EGFR DEL	1	5	0	0	0	1.00	1.00	1.00	1.00	1.00	1	22	0	0	0	1.00	1.00	1.00	1.00	1.00
EGFR G719	1	5	0	0	0	1.00	1.00	1.00	1.00	1.00	2	21	0	0	0	1.00	1.00	1.00	1.00	1.00
EGFR INS	0	6	0	0	0	1.00	-	1.00	-	1.00	2	21	0	0	0	1.00	1.00	1.00	1.00	1.00
EGFR L858R	1	5	0	0	0	1.00	1.00	1.00	1.00	1.00	3	20	0	0	0	1.00	1.00	1.00	1.00	1.00
EGFR L861Q	0	6	0	0	0	1.00	-	1.00	-	1.00	0	23	0	0	0	1.00	-	1.00	-	1.00
EGFR S768I	1	5	0	0	0	1.00	1.00	1.00	1.00	1.00	3	20	0	0	0	1.00	1.00	1.00	1.00	1.00
EGFR T790M	1	5	0	0	0	1.00	1.00	1.00	1.00	1.00	1	22	0	0	0	1.00	1.00	1.00	1.00	1.00
ERBB2	0	6	0	0	0	1.00	-	1.00	-	1.00	0	23	0	0	0	1.00	-	1.00	-	1.00
KRAS G12C	1	5	0	0	0	1.00	1.00	1.00	1.00	1.00	1	22	0	0	0	1.00	1.00	1.00	1.00	1.00
MET	0	6	0	0	0	1.00	-	1.00	-	1.00	1	22	0	0	0	1.00	1.00	1.00	1.00	1.00
NTRK	0	6	0	0	0	1.00	-	1.00	-	1.00	0	23	0	0	0	1.00	-	1.00	-	1.00
RET	1	5	0	0	0	1.00	1.00	1.00	1.00	1.00	2	21	0	0	0	1.00	1.00	1.00	1.00	1.00
ROS1	1	5	0	0	0	1.00	1.00	1.00	1.00	1.00	2	21	0	0	0	1.00	1.00	1.00	1.00	1.00

20/10 ng																				
	Reference										Biological									
call	TP	TN	FP	FN	Excluded	Accuracy	PPA	NPA	PPV	NPV	TP	TN	FP	FN	Excluded	Accuracy	PPA	NPA	PPV	NPV
ALK	1	5	0	0	0	1.00	1.00	1.00	1.00	1.00	1	20	0	1 [a]	1	0.95	0.50	1.00	1.00	0.95
BRAF V600E	4	2	0	0	0	1.00	1.00	1.00	1.00	1.00	0	23	0	0	0	1.00	-	1.00	-	1.00
EGFR DEL	1	5	0	0	0	1.00	1.00	1.00	1.00	1.00	1	22	0	0	0	1.00	1.00	1.00	1.00	1.00
EGFR G719	1	5	0	0	0	1.00	1.00	1.00	1.00	1.00	2	21	0	0	0	1.00	1.00	1.00	1.00	1.00
EGFR INS	0	6	0	0	0	1.00	-	1.00	-	1.00	2	21	0	0	0	1.00	1.00	1.00	1.00	1.00
EGFR L858R	1	5	0	0	0	1.00	1.00	1.00	1.00	1.00	3	20	0	0	0	1.00	1.00	1.00	1.00	1.00
EGFR L861Q	0	6	0	0	0	1.00	-	1.00	-	1.00	0	23	0	0	0	1.00	-	1.00	-	1.00
EGFR S768I	1	5	0	0	0	1.00	1.00	1.00	1.00	1.00	3	20	0	0	0	1.00	1.00	1.00	1.00	1.00
EGFR T790M	1	5	0	0	0	1.00	1.00	1.00	1.00	1.00	1	22	0	0	0	1.00	1.00	1.00	1.00	1.00
ERBB2	0	6	0	0	0	1.00	-	1.00	-	1.00	0	23	0	0	0	1.00	-	1.00	-	1.00
KRAS G12C	1	5	0	0	0	1.00	1.00	1.00	1.00	1.00	1	22	0	0	0	1.00	1.00	1.00	1.00	1.00
MET	0	6	0	0	0	1.00	-	1.00	-	1.00	1	21	0	0	1	1.00	1.00	1.00	1.00	1.00
NTRK	0	6	0	0	0	1.00	-	1.00	-	1.00	0	22	0	0	1	1.00	-	1.00	-	1.00
RET	1	5	0	0	0	1.00	1.00	1.00	1.00	1.00	2	20	0	0	1	1.00	1.00	1.00	1.00	1.00
ROS1	1	5	0	0	0	1.00	1.00	1.00	1.00	1.00	2	20	0	0	1	1.00	1.00	1.00	1.00	1.00

15/7.5 ng																				
	Reference										Biological									
call	TP	TN	FP	FN	Ex-cluded	Accu-racy	PPA	NPA	PPV	NPV	TP	TN	FP	FN	Ex-cluded	Accu-racy	PPA	NPA	PPV	NPV
ALK	1	5	0	0	0	1.00	1.00	1.00	1.00	1.00	2	19	0	0	2	1.00	1.00	1.00	1.00	1.00
BRAF V600E	4	2	0	0	0	1.00	1.00	1.00	1.00	1.00	0	23	0	0	0	1.00	-	1.00	-	1.00

EGFR DEL	1	5	0	0	0	1.00	1.00	1.00	1.00	1.00	1	22	0	0	0	1.00	1.00	1.00	1.00	1.00
EGFR G719	1	5	0	0	0	1.00	1.00	1.00	1.00	1.00	2	20	0	0	1	1.00	1.00	1.00	1.00	1.00
EGFR INS	0	6	0	0	0	1.00	-	1.00	-	1.00	2	20	0	0	1	1.00	1.00	1.00	1.00	1.00
EGFR L858R	1	5	0	0	0	1.00	1.00	1.00	1.00	1.00	3	20	0	0	0	1.00	1.00	1.00	1.00	1.00
EGFR L861Q	0	6	0	0	0	1.00	-	1.00	-	1.00	0	22	0	0	1	1.00	-	1.00	-	1.00
EGFR S768I	1	5	0	0	0	1.00	1.00	1.00	1.00	1.00	3	20	0	0	0	1.00	1.00	1.00	1.00	1.00
EGFR T790M	1	5	0	0	0	1.00	1.00	1.00	1.00	1.00	1	22	0	0	0	1.00	1.00	1.00	1.00	1.00
ERBB2	0	6	0	0	0	1.00	-	1.00	-	1.00	0	22	0	0	1	1.00	-	1.00	-	1.00
KRAS G12C	1	5	0	0	0	1.00	1.00	1.00	1.00	1.00	0	22	0	0	1	1.00	-	1.00	-	1.00
MET	0	6	0	0	0	1.00	-	1.00	-	1.00	1	20	0	0	2	1.00	1.00	1.00	1.00	1.00
NTRK	0	6	0	0	0	1.00	-	1.00	-	1.00	0	21	0	0	2	1.00	-	1.00	-	1.00
RET	1	5	0	0	0	1.00	1.00	1.00	1.00	1.00	2	19	0	0	2	1.00	1.00	1.00	1.00	1.00
ROS1	1	5	0	0	0	1.00	1.00	1.00	1.00	1.00	2	19	0	0	2	1.00	1.00	1.00	1.00	1.00

10/5 ng																				
	Reference										Biological									
call	TP	TN	FP	FN	Excluded	Accuracy	PPA	NPA	PPV	NPV	TP	TN	FP	FN	Excluded	Accuracy	PPA	NPA	PPV	NPV
ALK	1	4	0	0	1	1.00	1.00	1.00	1.00	1.00	0	13	0	2 [b]	8	0.87	0.00	1.00	-	0.87
BRAF V600E	4	2	0	0	0	1.00	1.00	1.00	1.00	1.00	0	23	0	0	0	1.00	-	1.00	-	1.00
EGFR DEL	1	5	0	0	0	1.00	1.00	1.00	1.00	1.00	1	22	0	0	0	1.00	1.00	1.00	1.00	1.00
EGFR G719	1	5	0	0	0	1.00	1.00	1.00	1.00	1.00	2	18	0	0	3	1.00	1.00	1.00	1.00	1.00
EGFR INS	0	6	0	0	0	1.00	-	1.00	-	1.00	3	17	0	0	3	1.00	1.00	1.00	1.00	1.00
EGFR L858R	1	5	0	0	0	1.00	1.00	1.00	1.00	1.00	3	20	0	0	0	1.00	1.00	1.00	1.00	1.00
EGFR L861Q	0	6	0	0	0	1.00	-	1.00	-	1.00	0	20	0	0	3	1.00	-	1.00	-	1.00

EGFR S768I	1	5	0	0	0	1.00	1.00	1.00	1.00	1.00	3	20	0	0	0	1.00	1.00	1.00	1.00	1.00
EGFR T790M	1	5	0	0	0	1.00	1.00	1.00	1.00	1.00	1	22	0	0	0	1.00	1.00	1.00	1.00	1.00
ERBB2	0	6	0	0	0	1.00	-	1.00	-	1.00	0	20	0	0	3	1.00	-	1.00	-	1.00
KRAS G12C	1	5	0	0	0	1.00	1.00	1.00	1.00	1.00	0	20	0	0	3	1.00	-	1.00	-	1.00
MET	0	5	0	0	1	1.00	-	1.00	-	1.00	1	14	0	0	8	1.00	1.00	1.00	1.00	1.00
NTRK	0	5	0	0	1	1.00	-	1.00	-	1.00	0	15	0	0	8	1.00	-	1.00	-	1.00
RET	1	4	0	0	1	1.00	1.00	1.00	1.00	1.00	1	14	0	0	8	1.00	1.00	1.00	1.00	1.00
ROS1	1	4	0	0	1	1.00	1.00	1.00	1.00	1.00	0	15	0	0	8	1.00	-	1.00	-	1.00

[a] CC00-02 55 ALK fusion reads on TSO500

[b] CC00-01 40 ALK reads fusion and CC00-02 55 ALK fusions reads on TSO 500

Table S4. **Contrived reference sample concordance data reported for the TSO500 Assay at 100 ng input vs. the HDPCR NSCLC Panel for DNA and RNA.** True Positive (TP), True Negative (TN), False Positive (FP), Fasle Negative (FN), Positive Percent Agreement (PPA), Positive Predictive Value (PPV), Negative Predictive Value (NPV), and Negative Percent Agreement (NPA). Count cutoffs for HDPCR are listed in Table S2. Wells excluded did not meet the IC requirements for NSCLC HDPCR (50 IC counts Well 1/Well 3 and 100 IC counts Well 2). DNA samples are tested across two wells, with five targets in each well (Well 1 and Well 2).

					DNA					
Input	TP	TN	FP	FN	Excluded	Accuracy	PPA	NPA	PPV	NPV
40 ng	10	50	0	0	0	1.00	1.00	1.00	1.00	1.00
20 ng	10	50	0	0	0	1.00	1.00	1.00	1.00	1.00
15 ng	10	50	0	0	0	1.00	1.00	1.00	1.00	1.00
10 ng	10	50	0	0	0	1.00	1.00	1.00	1.00	1.00
					RNA					
Input	TP	TN	FP	FN	Excluded	Accuracy	PPA	NPA	PPV	NPV
20 ng	3	27	0	0	0	1.00	1.00	1.00	1.00	1.00
10 ng	3	27	0	0	0	1.00	1.00	1.00	1.00	1.00
7.5ng	3	27	0	0	0	1.00	1.00	1.00	1.00	1.00
5 ng	3	22	0	0	5<br>(1 Samples)	1.00	1.00	1.00	1.00	1.00

Table S5 **Institutional Review Board Statement.** Samples from Lykos Lab are IRB exempt under IRB 223081.

IRB/Ethic Committee Name	Protocol/IRB #	Approval Date
BioChain Institute Inc. IRB #1—Biomedical	IRB00008283	6/9/21
Vanderbilt University IRB	IRB 010294	10/15/22
Advarra IRB	CR00425931	3/6/23
	Pro00051469	3/6/23
